# Ototoxicity in Cancer Therapies

**DOI:** 10.1111/odi.70046

**Published:** 2025-07-30

**Authors:** Sady Selaimen da Costa, Nathaniel S. Treister, Caio Eddie de Melo Alves

**Affiliations:** ^1^ Department of Ophthalmology and Otorhinolaryngology, Faculty of Medicine Universidade Federal do Rio Grande do Sul (UFRGS) Porto Alegre Brazil; ^2^ Service Otorhinolaryngology and Head and Neck Surgery Hospital De Clínicas De Porto Alegre (HCPA) Porto Alegre Brazil; ^3^ Division of Oral Medicine and Dentistry Brigham and Women's Hospital Boston USA; ^4^ Department of Oral Medicine, Infection and Immunity Harvard School of Dental Medicine Boston USA; ^5^ Postgraduate Program in Medicine: Surgical Sciences Universidade Federal do Rio Grande do Sul (UFRGS) Porto Alegre Brazil

**Keywords:** cancer therapies, hearing, ototoxicity

## 1 Introduction

According to estimates, around 20 million people per year are diagnosed with cancer, and this prevalence only tends to increase, with forecasts pointing to a 77% increase in new diagnoses by 2050, with the most common types of cancer being lung, breast, and colorectal (Ferlay et al. 2024). But due to increasingly effective treatments, the number of cured patients is increasing, increasing the need to recognize and better address possible treatment‐related side effects. Among them, ototoxicity stands out, with a great impact on cognition, mental health, and quality of life (Burstein et al. 2017).

**FIGURE 1:**
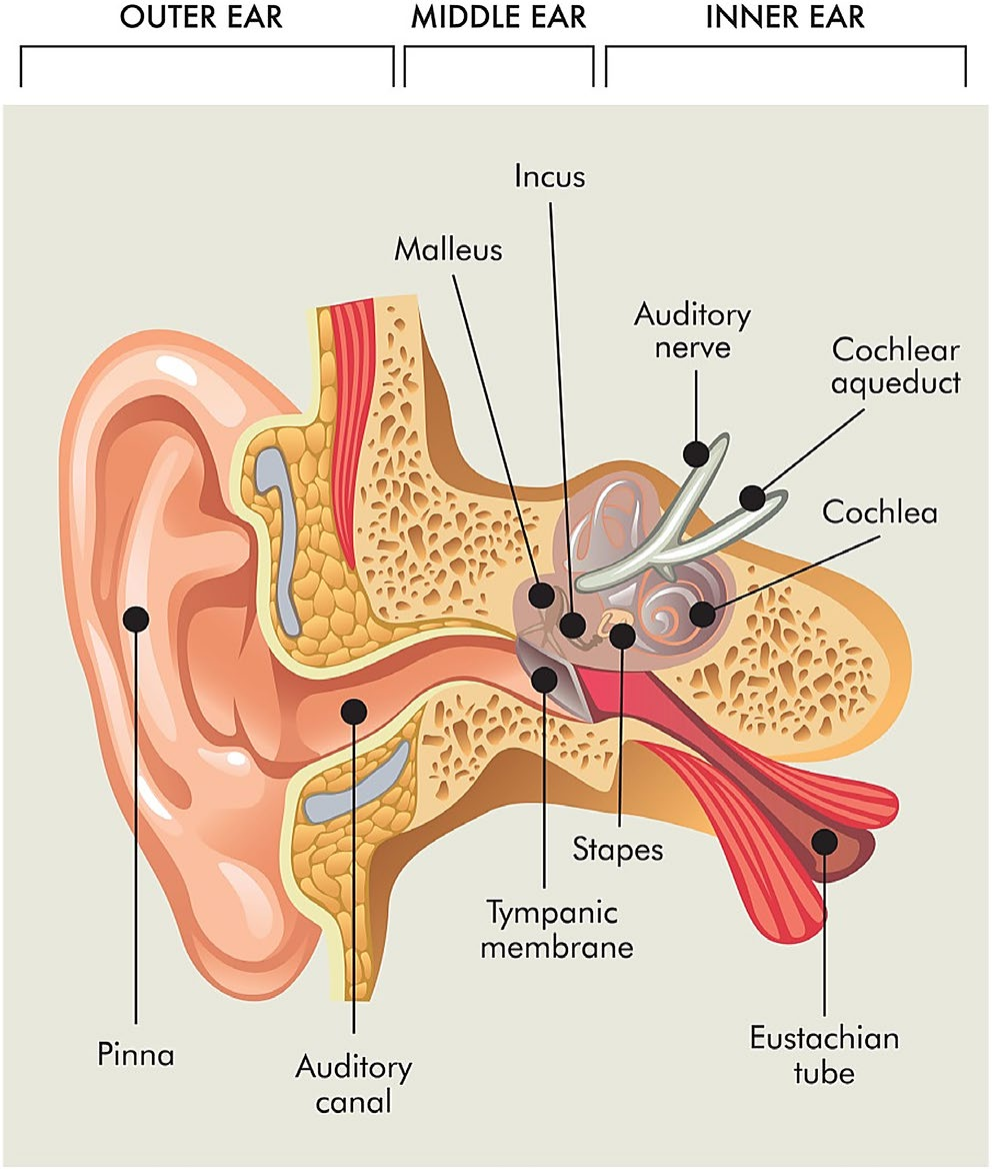
Anatomy of the human ear, where it is possible to observe the structures (middle ear, cochlea, auditory nerve and vestibular system) affected by ototoxic therapies (Landier 2016).

Ototoxicity is a phenomenon that refers to the undesirable side effects of certain therapies on the ear and the auditory nerve, which can affect both the auditory system and balance (Figure 1). These effects can lead to the degeneration of cochlear and/or vestibular tissue cells, resulting in their functional deterioration (Ganesan et al. 2018). In cancer patients, several treatments are potentially ototoxic, such as chemotherapy, radiotherapy, ear surgeries, and other commonly used medications, such as aminoglycoside antibiotics and loop diuretics (Patatt et al. 2022).

Cochleotoxicity can result in tinnitus, ear fullness, and/or hearing loss. Vestibulotoxicity can result in vertigo, dizziness, imbalance, and oscillopsia. Neurotoxicity may be a mechanism for the generation of tinnitus and may impact central auditory processing (Watts 2019). All of this leads to losses in communication and social interaction, with a negative impact on quality of life (Heinemann et al. 2020). In children, hearing loss often leads to delayed speech and language development, impairing literacy and educational performance (Yong et al. 2020). In adults, hearing loss has been associated with impaired communication, lower income, worse quality of life, and mental health, and cognitive impairment (Dillard et al. 2022).

The presentation of these symptoms is quite variable, and can be unilateral or bilateral, with rapid or more gradual onset, and may be reversible or permanent, and with mild severity to more important symptoms, such as profound deafness (Cianfrone et al. 2011).

## 2 Prevalence of Ototoxicity in Cancer Treatment

Assessing the incidence and prevalence of ototoxicity is not simple. Comparison between studies is not always possible due to methodological differences, differences in exposure to the specific medication, population differences, and detection protocols. This is likely why the reported prevalence of ototoxicity in patients receiving potentially ototoxic therapy varies widely from 4% to 90% (Landier 2016). For example, with cisplatin, ototoxicity affects 23%–50% of adults and up to 60% of children, but studies demonstrate elevated auditory thresholds in up to 100% of cancer patients treated with this drug (Ganesan et al. 2018).

In addition, factors that are known to influence the severity of ototoxicity include type of medication, route of administration, dose and period of treatment, age, sex, comorbid conditions (e.g., congestive heart failure, renal failure, hypertension), genetic susceptibility, geographic factors, noise exposure, and pre‐existing hearing loss (Bisht and Bist 2011). In particular, there is an increase in ototoxicity in very young children and in those receiving higher cumulative doses of ototoxic agents (Nitz et al. 2013).

## 3 Mechanisms of Ototoxicity

### 3.1 Toxicity Related to Chemotherapy and Medication

Chemotherapy and supportive care therapies (e.g., aminoglycoside antibiotics) used in the management of cancer patients can adversely affect the ear and vestibulocochlear nerve. Platinum‐based chemotherapy drugs are the most cited ototoxic agents, such as cisplatin, carboplatin, and oxaliplatin, often being used in combination (Oliveira et al. 2016). Of these medications, cisplatin is the most used, due to its high effectiveness, relatively low cost, and accessibility. First approved in the late 1970s, it is currently used to treat a wide variety of solid tumors involving the head and neck, lung, ovary, testicle, and bladder. In children, it can be used to treat neuroblastoma, osteosarcoma, hepatoblastoma, germ cell, and central nervous system tumors (Ding et al. 2012).

The frequency of cisplatin‐induced ototoxicity ranges from 45% to 83.3% when used as a single agent (Karasawa and Steyger 2015). Its mechanism of action, similar to that of aminoglycoside antibiotics, involves the generation of toxic levels of reactive oxygen species (ROS) within the cochlea. This leads to the destruction of cochlear hair cells and damage to the stria vascularis and spiral ganglion cells, initially affecting the basal turn of the cochlea, where high‐frequency sounds are processed (Ding et al. 2012). This ototoxicity causes bilateral, symmetrical, and irreversible sensorineural hearing loss, worse at high frequencies (4–8 kHz) and may also be associated with tinnitus. The degree of hearing loss is dose‐dependent, especially if greater than 400 mg/m^2^, although dosages as low as 200 mg/m^2^ of cisplatin have demonstrated ototoxicity (Sakat et al. 2019). Younger age (< 5 years) at the time of therapy, diagnosis of a central nervous system tumor, decreased renal function, rapid intravenous administration, and treatment with multiple potentially ototoxic agents also increase the risk of ototoxicity (Langer et al. 2013). The other platinum‐based chemotherapy agents differ in their chemical structure and adverse effect profiles. Carboplatin is generally less ototoxic than cisplatin (16.6%–75%), although the risk increases substantially when this agent is used in infants. Ototoxicity related to oxaliplatin is rare (Ruggiero et al. 2013).

Loop diuretics can also be ototoxic, especially when associated with concurrent chemotherapy. Generally, hearing loss is transient and occurs due to changes in fluid and electrolyte concentrations in the inner ear, which can result in edema of the cochlear tissue and an associated decrease in endocochlear potential (Rybak 1993).

### 3.2 Radiation‐Related Toxicity

Radiotherapy is used to treat many tumors of the central nervous system and other structures in the head, such as rhabdomyosarcoma and nasopharyngeal carcinoma, and can be used alone or as an adjuvant treatment before and after surgery. However, it is known to be associated with ototoxicity, with multifactorial etiology (Warrier et al. 2012). It is believed to be related to direct damage to the cochlear system, damaging the organ of Corti and atrophying the vestibulocochlear nerve, or damage to small vessels, leading to hypoxia of the inner ear structures. Possible radiation damage to the brain stem may also indirectly contribute to this hearing loss (Jovem and Lu 2001).

This toxicity is dose‐dependent, with doses greater than 30 Grays (Gy) of radiation to the posterior nasopharynx and mastoid region being associated with an increased risk of developing sensorineural hearing loss, serous otitis media, and associated conductive hearing loss (Landier 2016). The probability of hearing loss between 30 and 40 Gy is approximately 27% (Huang et al. 2023). Irradiation involving the external auditory canal can lead to a greater number of soft tissue infections and an increase in the production of earwax, further contributing to compromised hearing (Landier 2016).

The combined use of radiotherapy with chemotherapy presents a higher frequency of sensorineural hearing loss compared to patients treated with radiotherapy alone, especially for high‐frequency sounds (Low et al. 2006). This radiation‐related sensorineural hearing loss is generally permanent and progressive, and may begin during the acute phase of treatment or several years after its completion (Mujica‐Mota et al. 2013). An increased risk of sensorineural hearing loss has also been reported in male patients aged over 50 years and associated with post‐radiotherapy otitis media (Bhandare et al. 2007).

### 3.3 Surgery‐Related Toxicity

Tumors located within or near auditory structures or the auditory nerve, such as nasopharyngeal, parameningeal, vestibular, and skull base tumors and tumors affecting the temporal bone, can cause damage due to direct infiltration of these regions (Guillaume et al. 2012).

Depending on the diagnosis and location, the planned surgical treatment may lead to potential hearing or vestibular losses. Patients with tumors of the central nervous system may also experience these losses due to rapid changes in intracranial pressure and variation in cerebrospinal fluid associated with lumbar puncture, tumor resection, or ventriculostomy (Wang et al. 2013).

### 3.4 Genetic Predisposition

Patients undergoing the same treatment known to be ototoxic may present a widely varying prevalence and severity, with some patients remaining unaffected at high cumulative doses, while others suffer severe damage at low doses. Among several possible factors, genetic predisposition may explain these differences (Ross et al. 2009).

Several genes related to antioxidant regulation, neurotransmission, or auditory function have been associated with increased risk of ototoxicity, including ACYP2, LRP2, TPMT, SOD2, and COMT (Thiesen et al. 2017). Of these, ACYP2, which encodes acylphosphatase‐2 expressed in the cochlea that hydrolyzes phosphoenzymatic intermediates of membrane pumps that affect Ca^2+^ ion homeostasis, has the highest correlation with cisplatin ototoxicity (Xu et al. 2015).

Nguyen and Jeyakumar (2019), in a literature review, showed that all mutations associated with aminoglycoside‐induced ototoxicity were mitochondrial. The mitochondrial 12S rRNA A1555G mutation was identified as the primary genetic factor underlying hearing loss in these cases, and it was found among individuals of American, Chinese, Arab‐Israeli, Spanish, and Mongolian descent. The second most frequently identified mutation was C1494T.

Genetics offers a promising path for investigation; however, there is currently no robust and consistent evidence that any specific gene constitutes a definitive causal factor. At this stage, such genetic variations should be considered susceptibility factors rather than absolute predictors. Therefore, a balanced and integrative approach to patient care is essential (Iațentiuc et al. 2025).

## 4 Diagnosis of Ototoxicity

**FIGURE 2:**
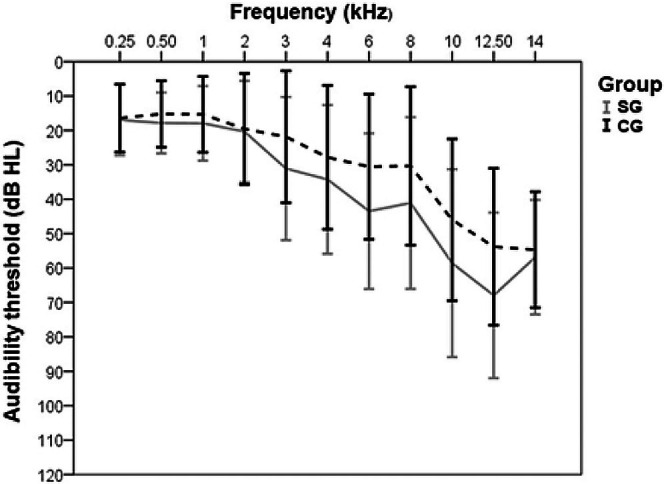
Comparison of the means and standard deviations of the pure‐tone auditory thresholds, in dB, obtained in the pure‐tone threshold audiometry between patients treated for head and neck cancer submitted to chemotherapy and/or radiotherapy (Group SG) and control group (Group CG) (Fukazawa et al. 2020).

The standard method for diagnosing ototoxicity is pure‐tone audiometry, and whenever possible, covering high frequencies (Le Prell et al. 2022) (Figure 2). It can be performed on both adults and children, generally those over 5 years old. When younger, audiometry can be done in an adapted way through playful techniques or visual reinforcement (Bass and Bhagat 2014). Brainstem auditory evoked potentials are another diagnostic option, especially when used in children or uncooperative patients. Distortion product otoacoustic emissions should also be requested, as they usually help with early diagnosis, due to their high sensitivity to cochlear damage (Knight et al. 2007).

There are several audiometric classifications of hearing loss due to ototoxicity, but there is still no consensus on which should be used as a standard. These different classifications result in variations in the prevalence of the diagnosis and the degree of involvement (Patatt et al. 2022). With most scales, hearing loss receives a score ranging from 0 (normal hearing or clinically insignificant loss) to 4 (severe or profound hearing loss) and can be used in adults and children (Konrad‐Martin et al. 2014). The most used classifications for children are Chang and SIOP, and for adults, ASHA and CTCAE (Waissbluth et al. 2017).

The recommended frequency of hearing tests is variable and depends on multiple factors such as age, comorbidities, previous hearing loss, and the cancer therapy modality. The minimum recommended is an otorhinolaryngological evaluation and a hearing test to be carried out before starting treatment and at the end of it, but patients treated with platinum‐based therapies should also be evaluated in the long‐term follow‐up setting, even in the absence of hearing complaints (> 2 years) (Ganesan et al. 2018).

Children, due to their greater risk of ototoxicity and greater potential for future harm, should undergo hearing monitoring every 1–2 courses of platinum‐based chemotherapy and during long‐term follow‐up, to minimize damage through early detection, auditory intervention, and, if possible, modification of ototoxic therapy (Bass and Bhagat 2014).

Patients who have received radiotherapy to the head or ear should be audiologically monitored every 5 years, or earlier if indicated, due to the potential risk of progressive hearing loss over the years (Mujica‐Mota et al. 2013).

## 5 Prophylactic Measures

Preventing hearing loss begins by choosing the therapy to be instituted. Whenever possible, it is optimal to avoid or limit exposure to known ototoxic therapies and medications. Research efforts are ongoing to identify new treatments with fewer side effects (Dillard et al. 2021).

Before starting cancer therapy, an assessment of the ototoxicity risk profile must be carried out, taking into account factors such as exposure to intense noise, drug combination, previous hearing loss, liver or kidney problems, administration in children/elderly, and pregnancy. In the presence of one of these factors, the risk of hearing loss is greater than 50% (Hyppolito and Oliveira 2005). Likewise, advances in genetic screening tests to identify individuals susceptible to ototoxicity are being investigated and may help in the future (Ganesan et al. 2018).

There has been great interest in evaluating potential otoprotective agents to protect against the anticipated damaging effects of therapy. The most investigated in clinical trials to date include sodium thiosulfate, amifostine, and N‐acetylcysteine (Katzenstein et al. 2009). Systemic administration of these agents, however, may also result in a reduction of antitumor efficacy. Transtympanic administration of otoprotective agents (mainly agents with antioxidant activity such as N‐acetylcysteine and corticosteroids) has been evaluated, but still without results with statistical relevance (Rolland et al. 2019). No agents are currently approved as otoprotectors, and further randomized trials are needed to determine efficacy as well as to establish the ideal dose and duration of the otoprotective agent (Dillard et al. 2022).

Regarding radiotherapy, to reduce ototoxicity risk, it is recommended that the total dose not exceed 30 Gy, and an associated hypofractionation scheme can be used to further reduce this risk. In the case of radiotherapy for the treatment of a vestibular schwannoma, for example, a total prescribed dose of 21–30 Gy in 3–7 Gy fractions can be used for 3–10 days (Bhandare et al. 2010). Regarding the type of radiotherapy, ototoxicity associated with intensity‐modulated radiotherapy was less common than conventional radiotherapy, and stereotactic radiotherapy appears to be a better option for hearing protection than radiosurgery (Huang et al. 2023).

In addition to pharmacological interventions, non‐pharmacological strategies are essential for a more comprehensive approach to preventing ototoxicity. Lifestyle modifications, such as avoiding loud noise exposure, abstaining from smoking, and ensuring proper nutritional intake, particularly antioxidants like vitamins C and E and magnesium, have shown potential to mitigate oxidative stress in the cochlea and preserve hearing function (Natarajan et al. 2023).

Furthermore, audiologists play a central role in ototoxicity prevention by implementing baseline and serial audiometric evaluations. This allows for the early identification of auditory damage and timely intervention through dose adjustments or treatment changes (Bass and Bhagat 2014) (Table 1).

**TABLE 1:** Summary of cancer treatment therapies.

Cancer treatment therapies	Mechanism of action of its ototoxicity	Prevention
Chemotherapy and supportive care therapies	Production of reactive oxygen species in the cochlea at toxic levels	Avoid or limit exposure to known ototoxic therapies and medications. When not possible, avoid using them in combination or in doses or for a prolonged period
Radiation	Direct damage to the cochlear system, vascular system, and/or brain stem	Hypofractionation schemes, associated with reduced total radiation dose
Surgery	Resections or manipulations of tumors related to the ear or auditory nerve	Individualized approach for each case, which can be combined with other types of treatment (Silva et al. 2023)

## 6 Rehabilitation of Ototoxicity

Despite otoprotective measures, many at‐risk patients will develop permanent auditory sequelae with varied impacts on their quality of life. Patients with hearing loss and communication difficulties should be evaluated for auditory rehabilitation, which can be achieved through amplification devices, cochlear implants, and hearing aids, in conjunction with communicative strategies (Ganesan et al. 2018). In children, even mild hearing losses can lead to major long‐term impacts, such as delays in speech and cognitive development, as well as difficulties in school and social performance. Therefore, interventional measures must be taken early (Cianfrone et al. 2011).

Patients undergoing radiation and developing middle ear effusion may undergo tympanotomy as a form of treatment. Hyperbaric oxygen therapy is also a treatment option for most individuals with effusion (Huang et al. 2023).

In addition to conventional hearing aids and cochlear implants, auditory training programs and speech therapy sessions have been effective in enhancing communication skills in patients with hearing impairment due to ototoxicity. These non‐device‐based strategies improve auditory processing and compensatory skills, especially in pediatric and elderly populations (Cianfrone et al. 2011).

New frontiers in rehabilitation also include experimental regenerative therapies. Gene therapy, stem cell transplantation, and molecular approaches targeting hair cell regeneration are under investigation and offer promise for reversing or minimizing cochlear damage in the long term (Kros and Steyger 2019).

## 7 Conclusion

Ototoxicity is a clinically relevant adverse effect of most cancer therapies, including radiotherapy, chemotherapy, and ear‐related surgeries. Damage to cochlear and vestibular cells can lead to hearing and balance disorders, such as vertigo, tinnitus, and hearing loss, which may significantly affect emotional well‐being, social functioning, and, in children, neurocognitive development and academic performance.

Given these effects, it is essential to adopt broad preventive and rehabilitative strategies. This includes early risk stratification, ongoing audiological monitoring, the use of otoprotective agents whenever available, and timely rehabilitative interventions. These may involve hearing aids, cochlear implants, auditory training, or speech therapy.

Looking to the future, research should prioritize the development of otoprotective agents that can effectively prevent ototoxicity without compromising antitumor efficacy. In particular, studies on genetic biomarkers that indicate individual susceptibility to ototoxicity could support the creation of personalized prevention protocols in oncology. Non‐pharmacological strategies, such as antioxidant‐rich nutritional interventions, lifestyle changes, and standardized audiological monitoring protocols, should also be systematically tested in clinical trials to confirm their effectiveness and expand the range of available therapies.

In the end, protecting a patient's hearing and balance goes beyond medical treatment. It is about preserving essential aspects of everyday life—like communication, safe mobility, and social connection—and ensuring that even during or after such a challenging journey as cancer treatment, the person can live with dignity and quality of life.

## Author Contributions

**Sady Selaimen da Costa:** writing – original draft, writing – review and editing, conceptualization, investigation, methodology, formal analysis, project administration, supervision, data curation. **Nathaniel S. Treister:** writing – review and editing, formal analysis, data curation, supervision, conceptualization, methodology, project administration. **Caio Eddie de Melo Alves:** conceptualization, investigation, writing – original draft, methodology, writing – review and editing, formal analysis, project administration, supervision, data curation.

## Conflicts of Interest

The authors declare no conflicts of interest.

## Data Availability

Data sharing is not applicable to this article as no new data were created or analyzed in this study.
